# Prospective Association between Whole Grain Consumption and Hypertension: The Furukawa Nutrition and Health Study

**DOI:** 10.3390/nu12040902

**Published:** 2020-03-26

**Authors:** Ikuko Kashino, Masafumi Eguchi, Takako Miki, Takeshi Kochi, Akiko Nanri, Isamu Kabe, Tetsuya Mizoue

**Affiliations:** 1 Department of Epidemiology and Prevention, Center for Clinical Sciences, National Center for Global Health and Medicine, Tokyo 162-8655, Japan; takakomiki-tky@umin.ac.jp (T.M.); nanri@fwu.ac.jp (A.N.); mizoue@hosp.ncgm.go.jp (T.M.); 2 Department of Health Administration, Furukawa Electric Corporation, Tokyo 100-8322, Japan; masafumi.eguchi@furukawaelectric.com (M.E.); takeshi.kochi@furukawaelectric.com (T.K.); isamu.kabe@kubota.com (I.K.); 3 Department of Food and Health Sciences, International College of Arts and Sciences, Fukuoka Women’s University, Fukuoka 813-8529, Japan

**Keywords:** blood pressure, hypertension, whole grain, Asians, Japan, cohort study, epidemiology

## Abstract

Hypertension has become a major public health issue worldwide. Whole grains contain higher levels and a broader range of nutrients with potential health benefits and may decrease the risk of hypertension. However, no prospective studies have investigated this association in the high-income Asia Pacific region, which has the lowest whole grain intake worldwide. Thus, we examined the prospective association between whole grain consumption and the development of hypertension in Japan. Participants included 944 working Japanese adults aged 19–68 years who had no hypertension at baseline and completed a 3-year follow-up survey. Whole grain consumption was assessed via a self-administered dietary questionnaire. Multivariate logistic regression analysis was carried out to examine the association between whole grain consumption and hypertension, adjusting for potential confounding factors, such as sociodemographic, lifestyle, dietary, and occupational characteristics. After 3 years, 9.4% (86 cases) of the study participants had developed hypertension. More frequent whole grain consumption, classified as an intake frequency of “sometimes or always”, was associated with lower odds of hypertension (multivariate-adjusted odds ratio: 0.36; 95% confidence interval: 0.16–0.83; *p* for trend = 0.04) compared with no consumption. Consuming more whole grains may decrease the risk of developing hypertension.

## 1. Introduction

With the increasing number of hypertensive individuals, hypertension has become a major public health issue around the world and results in death and disability via many different causes [1]. In Japan, deaths associated with blood pressure regardless of age or sex increased rapidly between 1990 and 2015 compared with those in western or other high-income Asia-Pacific countries [1]. Although modifiable risk factors associated with hypertension are well known, including unhealthy diets (e.g., excessive salt consumption, high levels of saturated and trans fats, and low intake of fruits and vegetables), physical inactivity, alcohol and tobacco use, and being overweight or obese [2], additional approaches for preventing hypertension should also be explored.

Whole grains, including brown rice, contain more abundant and diverse nutrients with potential health benefits (e.g., fiber, vitamins, and minerals) compared with refined grains, such as white rice. A meta-analysis of four cohort studies conducted in the U.S. showed that whole grain intake was inversely associated with the risk of hypertension [3]. In 2015, the US Dietary Guideline Advisory Committee recommended replacing most refined grains with whole grains in an effort to improve dietary quality among Americans [4]. However, there have been no prospective studies conducted in high-income Asia-Pacific countries including Japan, which has the lowest whole grain intake in the world [5]. Additional evidence from Asia is thus crucial for enhancing the generalizability of the association between hypertension and whole grain intake.

To this end, we prospectively examined the association between dietary habit of whole grain intake and hypertension risk in the Japanese population.

## 2. Materials and Methods

### 2.1. Study Procedure

In the Furukawa Nutrition and Health Study, which is part of the Japan Epidemiology Collaboration on Occupational Health Study, factory workers were recruited from two work sites of the same company in Chiba and Kanagawa Prefectures to participate in a survey that was carried out as part of a routine health exam. The survey was conducted both at baseline and 3 years later (April 2012 and 2015 at the work site in Chiba; and May 2013 and 2016 in Kanagawa), as previously described [6]. Prior to these health exams, all employees (*n* = 2828) were invited to participate and asked to fill out study-specific questionnaires related to diet and general health-related lifestyle. On the day of the health checkup, research staff checked that the questionnaires had been completed and participants were asked to clarify answers if needed. Additional information obtained during the health exams, such as anthropometric and biochemical measurements and dietary history (e.g., alcohol habits), was also evaluated. Informed consent was obtained from the participants, and the study protocol was approved by the Ethics Committee of the National Center for Global Health and Medicine, Japan.

### 2.2. Participants

**Figure 1:**
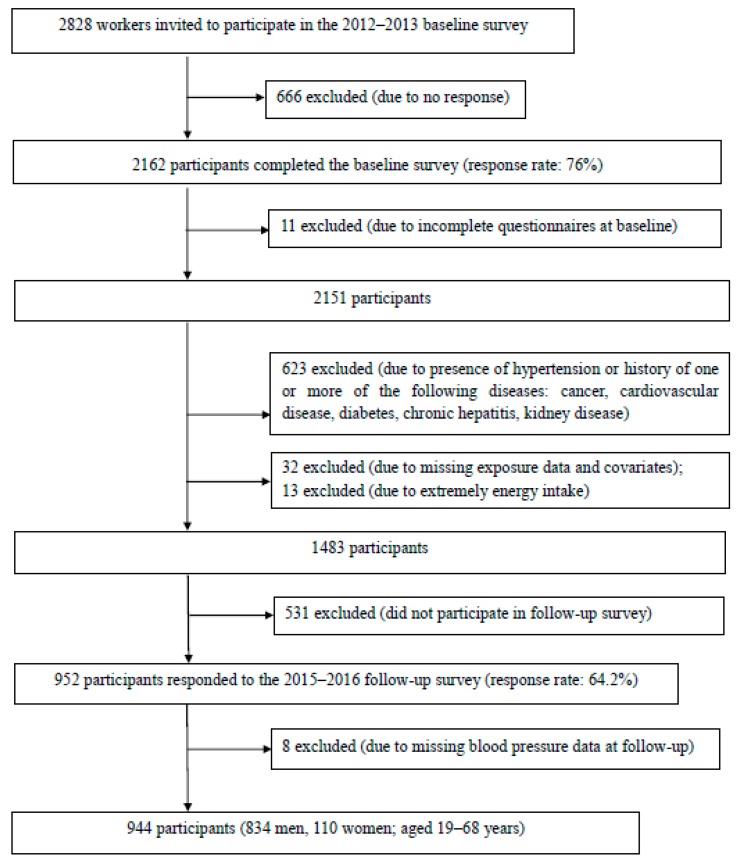
Flowchart of the study population.

Of 2828 workers partaking in the health checkups, 2162 (76%) agreed to participate in the baseline survey, of whom 2151 further completed the baseline questionnaire, including diet and covariate information such as lifestyle characteristics and disease history. Among these participants, we excluded 573 with hypertension and 50 with a history of one or more of the following conditions at baseline: cancer in 11, cardiovascular disease in 8, chronic hepatitis in 2, kidney disease including nephritis in 2 and pancreatitis in 1, and diabetes in 29. We further excluded 32 participants with missing data on covariates and 13 with extreme total energy intake at baseline (i.e., greater than the mean ± 3 standard deviations). Of the remaining 1483 participants, 952 responded to the 3-year follow up survey (response rate: 64.2%). An additional 8 participants with missing blood pressure data at follow-up were excluded from the analysis. In total, 944 participants (834 men and 110 women aged 19–68 years) were analyzed as part of this study (Figure 1). 

### 2.3. Definition of Hypertension

Systolic/diastolic blood pressure was measured using an automated sphygmomanometer (BP 103i II for the 2012 and 2015 survey and HEM-907 for the 2013 and 2016 survey, Omron Health Care Co. Ltd., Kyoto, Japan). Participants underwent a 3-minute rest prior to blood pressure measurement at their work site in Chiba, whereas no such precondition was specified in Kanagawa. Hypertension was defined as systolic blood pressure ≥140 mm Hg and/or diastolic blood pressure ≥ 90 mm Hg or as use of an antihypertensive agent.

### 2.4. Assessment of Dietary Intakes

Dietary intake during the month prior to the health exam was evaluated using the Brief Self-administered Diet History Questionnaire; validation of the questionnaire was previously demonstrated [7,8]. The frequency of whole grain intake was assessed by the question “Do you eat brown rice, germinated rice, or a wheat/millet mix with rice?” Participants selected one of the following four answers: never, rarely, sometimes, or always. Intakes of foods and nutrients, such as rice, bread, noodles, vegetables, fruits, pulses, meats, dairy, soft drinks, sodium, and total energy, were estimated using an *ad hoc* computer algorithm adopted for the Brief Self-administered Diet History Questionnaire and the food composition table for Japan [9]. According to the validation study, Spearman’s correlation coefficients for energy-adjusted rice, bread, noodles, vegetables, fruits, pulses, meats, dairy, soft drinks, sodium, and total energy between dietary records and the Brief Self-administered Diet History Questionnaire were 0.66, 0.71, 0.49, 0.62, 0.61, 0.45, 0.67, 0.70, 0.39, 0.61, and 0.45 for women and 0.71, 0.65, 0.47, 0.62, 0.70, 0.55, 0.56, 0.70, 0.49, 0.60, and 0.40 for men, respectively [7,8].

### 2.5. Assessment of Other Health-Related Variables

Exertion of physical activity during work and housework or during commuting and leisure time were expressed as the sum of metabolic equivalent (MET) multiplied by duration of time (in hours) across physical activities with different levels. Body mass index (kg/m^2^) was calculated as body weight divided by height squared, which were measured to the nearest 0.1 kg and 0.1 cm, respectively, by trained staff. Participants’ answers to the health-related lifestyle questionnaire provided information on night or rotating shift work, overtime work, use of tobacco, alcohol consumption, and physical activity exertion during work, housework, and commuting and leisure time.

### 2.6. Statistical Analysis

To determine the potential bias due to the participants’ selection process in this study, we compared baseline characteristics among those who completed the 3-year follow-up survey and those who were lost to follow-up. Participants were divided into three groups according to their whole grain intake frequency: never, rarely, and sometimes, or always. The participants’ characteristics were evaluated according to frequency of whole grain intake, using linear regression analysis for the continuous variables and the Mantel–Haenszel test for trend for the categorical variables. 

We performed multiple logistic regression analysis to determine the association of intake of whole grains with the likelihood of developing hypertension, estimated as odds ratios (ORs) with 95% confidence intervals (CIs). We used two different models for the analysis and adjusted for potential confounding variables according to the literature [3] and our previous study [10]. Model 1 was adjusted for age, sex, and work site (Chiba vs. Kanagawa). Model 2 was further adjusted for smoking (never smoked, former smoker, current smoker of <20 cigarettes/day, or current smoker of ≥20 cigarettes/day), alcohol consumption (nondrinker, drinker consuming 1–3 days/month, weekly drinker consuming <1, 1 to <2, or ≥2 servings/day; one serving contains 23 g of ethanol), physical activity at work and housework or in commuting to work (<3, 3 to <7, 7 to <20, or ≥20 MET-hours/day), leisure-time physical activity (0, >0 to <3, 3 to <10, or ≥10 MET-hours/week), body mass index (kg/m^2^), night or rotating shift work (yes/no), overtime work (<10, 10 to <30, or ≥30 hours/month), total energy intake (kcal/day), and food and nutrient intakes (vegetables, fruits, pulses, meats, dairy, soft drinks, rice, bread, noodles, and sodium) expressed as energy densities. To determine linear trends, the three categories were assigned ordinal values (0–2) and treated as a continuous variable. Furthermore, to examine the association between frequency of repeated whole grain intake over time and development of hypertension, we defined the participants with a consistent whole grain intake frequency of “never” or “sometimes or always” between baseline and the 3-year follow-up as having maintained a low or high intake frequency, respectively. All statistical analyses were performed using the SAS statistical software package, version 9.4 for Windows (SAS Institute Inc., Cary, NC, USA). A two-sided *p* value < 0.05 was representative of statistical significance in all analyses.

## 3. Results

Participant characteristics according to frequency of whole grain intake are shown in Table 1. Approximately 20% of participants consumed whole grains either sometimes or always, while 56% of participants did not consume whole grains. Participants with frequent whole grain intake tended to consume more energy, fats, proteins, vegetables, fruits, pulses, and dairy but less carbohydrates, soft drinks, and rice compared with those with less frequent intake (*p* for trend <0.05 for all). In addition, participants with more frequent whole grain intake were less likely to be current smokers, physically active during work, or night or rotating shift workers but were more likely to be active during leisure time compared with those with less frequent whole grain intake. Compared with those who participated in the follow-up survey, those who did not participate in the follow-up survey were older and more likely to have overtime work and tended to consume lower amounts of pulses, while other characteristics (e.g., whole grain consumption) were similar between both groups (Supplementary Materials Table S1).

At the 3-year follow-up, 89 participants (9.4%) who were not hypertensive at baseline had developed hypertension. Based on the multiple logistic regression model 1, after adjusting for age, sex, and work site, participants that reported consuming whole grains either sometimes or always exhibited lower odds of developing hypertension compared with those who did not consume whole grains (Table 2). In model 2, after additional adjustments for lifestyle, work-related, and dietary factors, the adjusted ORs for developing hypertension were 1.00 (reference), 1.02 (95% CI: 0.57–1.83), and 0.36 (95% CI: 0.16–0.83) for the whole grain intake categories of “never”, “rarely”, and “sometimes or always”, respectively (*p* for trend = 0.04). A similar but somewhat stronger association was observed in an additional analysis using both baseline and follow-up dietary data (n = 513); the multivariable-adjusted OR of hypertension for those who maintained high consumption was 0.20 (0.05–0.73) compared with participants that maintained low whole grain consumption throughout the entire 3-year study period (Supplementary Materials Table S2).

## 4. Discussion

This is the first prospective study to address the association between whole grain consumption and hypertension in an Asian population. The results of this study demonstrated about 60% reduced likelihood of developing hypertension among those with a frequent (“sometimes or always”) consumption of whole grains compared with no consumption, even after adjusting for numerous potential confounders. These findings are supported by a recent meta-analysis of four cohort studies conducted in the U.S. [3]. In Iran, a cross-sectional study of 827 participants aged 18–74 years showed a 21% lower prevalence of hypertension among adults in the highest quartile compared with the lowest quartile of whole grain intake [11]. In Japan, a randomized controlled study reported that daily supplementation with rice bran, a component of brown rice and by-product of rice polishing, reduced systolic blood pressure in patients with mild hypertension and high-normal blood pressure [12]. The findings from this prospective observational study provide evidence to support the hypothesis that whole grain consumption lowers the risk for hypertension in Asian populations.

The mechanism underlying the beneficial effect of whole grain intake on the risk of hypertension remains unclear; however, data from several mechanistic and experimental studies have been reported. First, in an animal experiment, the potent antihypertensive peptide Leu-Arg-Ala from rice bran protein was shown to exhibit its effects on hypertension via endothelial nitric oxide-mediated vasorelaxation [13]. Second, dietary supplementation with either a Driselase or ethanol fraction of rice bran significantly decreased blood pressure in stroke-prone spontaneously hypertensive rats [14]. Third, a rat experiment suggested that the antihypertensive effect of virgin rice bran oil, which is extracted from the germ and inner husk of rice by the cold-press method, may be mediated by restored hemodynamics, increased nitric oxide bioavailability, and alleviated oxidative stress and inflammation [15]. Finally, injection of gamma-aminobutyric acid, which is commonly produced by germinated brown rice, enhanced the arterial baroreceptor reflex function and adjusted heart rate to reduce blood pressure fluctuations in spontaneously hypertensive rats [16]. Finally, high consumption of fiber rich in whole grain may prevent hypertension through change of gut microbiota [17].

The current study has several strengths including its prospective study design, relatively homogenous study population, and adjustments for a wide range of known and suspected risk factors for hypertension, including work-related factors and nutrients that exhibit a known link to hypertension. There were also some limitations associated with the present study. First, 35.8% of participants did not complete the 3-year follow-up survey; this may have introduced selection bias. However, baseline characteristics were generally similar between participants who completed follow-up and those lost to follow-up. Second, frequency of whole grain intake was ascertained using a single question with simple response options, which did not allow us to estimate the amount of whole grain intake. Third, the dietary measurements taken at baseline might not reflect long-term habitual consumption. Nevertheless, our secondary analysis, which considered both baseline and follow-up dietary data together, also indicated a similar strength of association compared to the primary analysis. Finally, because this study included only workers, the majority of which were men, the generalizability of the findings to the general population in Japan is unclear.

## 5. Conclusions

Results of this prospective study demonstrate an association between frequent whole grain intake and lower risk of developing hypertension among working individuals in Japan, strengthening the hypothesis that greater consumption of whole grains decreases the risk of hypertension.

## Figures and Tables

**Table 1:** Baseline characteristics of the study population according to frequency of whole grain intake.

	Frequency of Whole Grain Intake			
	Never	Rarely	Sometimes or Always	*p*_trend_^1)^
No. of participants	530	221	193	
Age, years (mean ± SD)	39.7 ± 8.7	39.4 ± 8.8	40.9 ± 8.0	0.18
Females, %	10.6	11.3	15.0	0.12
Work site (survey in April 2012), %	56.0	52.9	44.6	0.01
Smoking status (current), %	34.9	28.1	21.8	<0.001
Physical activity during work, housework, or commuting to work (≥20 METs-hours/day), %	28.3	19.5	12.4	<0.001
Leisure-time physical activity (≥10 METs-hours/week), %	24.9	30.3	34.2	0.01
Body mass index (mean ± SD), kg/m^2^	22.4 ± 2.8	23.0 ± 3.2	22.4 ± 2.6	0.02
Night or rotating shift work (yes), %	26.8	13.6	8.8	<0.001
Overtime work (≥30 hours/month), %	24.5	26.2	26.9	0.47
Alcohol consumption (current), % ^2)^	52.5	47.1	53.9	0.97
Dietary nutrient and food intake (mean ± SD)				
Total energy, kcal/day	1753 ± 484	1854 ± 482	1781 ± 444	0.02
Carbohydrate, % energy	55.3 ± 8.1	55.3 ± 7.4	53.6 ± 7.8	0.03
Fat, % energy	23.7 ± 5.7	24.1 ± 4.8	25.1 ± 5.6	0.01
Protein, % energy	13.4 ± 2.6	13.9 ± 2.3	14.2 ± 2.2	<0.001
Sodium, mg/1000 kcal	2300 ± 473	2322 ± 436	2282 ± 401	0.66
Vegetables, g/1000 kcal	108.5 ± 59.9	129.4 ± 69.4	138.4 ± 71.7	<0.001
Fruits, g/1000 kcal	36.4 ± 44.1	48.4 ± 48.7	51.5 ± 52.2	<0.001
Pulses, g/1000 kcal	24.2 ± 20.1	25.4 ± 17.5	29.7 ± 19.9	0.004
Meats, g/1000 kcal	39.9 ± 18.2	39.4 ± 18.5	38.8 ± 16.2	0.76
Dairy, g/1000 kcal	47.9 ± 51.8	48.1 ± 43.5	62.7 ± 53.0	0.002
Soft drinks, g/1000 kcal	52.1 ± 80.0	41.6 ± 51.0	32.2 ± 51.5	0.003
Rice, g/1000 kcal	186.3 ± 67.0	188.5 ± 60.3	172.8 ± 68.0	0.03
Bread, g/1000 kcal	22.5 ± 18.9	20.1 ± 15.0	22.0 ± 17.9	0.25
Noodles, g/1000 kcal	44.4 ± 31.5	43.6 ± 27.4	41.4 ± 26.8	0.49

Abbreviations: MET, metabolic equivalent. ^1)^ Based on the Mantel–Haenszel test for categorical variables and linear regression analysis for continuous variables. ^2)^ Alcohol consumption at least one day per week.

**Table 2:** Prospective associations between whole grain intake and hypertension.

	Frequency of Whole Grain Intake						
	Never		Rarely		Sometimes or Always		*p*_trend_^1)^
Participants (*n*)	530		221		193		
Cases (*n*)	57		24		8		
Adjusted odds ratios (95% CI)							
Model 1^2)^	1.00	(Reference)	1.04	(0.62–1.75)	0.35	(0.16–0.77)	0.02
Model 2^3)^	1.00	(Reference)	1.02	(0.57–1.83)	0.36	(0.16–0.83)	0.04

Abbreviations: CI, confidence interval. ^1)^ Linear trends across the whole grain intake categories were determined using each category as an ordinal variable. ^2)^ Model 1 adjusted for age (year, continuous), sex, and site (survey in April 2012 or in May 2013). ^3)^ Model 2 additionally adjusted for smoking (never smoked, former smoke, current smoker consuming <20 cigarettes/day, or current smoker consuming ≥20 cigarettes/day), alcohol consumption (nondrinker, drinker consuming 1–3 days/month, weekly drinker consuming <1, 1 to <2, or ≥2 serving/day; one serving contains 23 g of ethanol), physical activity at work and housework or in commuting to work (<3, 3 to <7, 7 to <20, or ≥20 MET-hour/day), leisure-time physical activity (not engaged, >0 to <3, 3 to <10, or ≥10 MET-hours/week), body mass index (kg/m^2^), night or rotating shift work (yes/no), overtime work (<10, 10 to <30, or ≥30 hours/month), total energy intake (kcal/day), and nutrient and food intake expressed as energy density (sodium, vegetables, fruits, pulses, meat, dairy, soft drinks, rice, bread, and noodles).

## Supplementary Materials

The following are available online at https://www.mdpi.com/2072-6643/12/4/902/s1, Table S1: Baseline characteristics of the participants who completed the follow-up survey versus those lost to follow-up, Table S2: Odds ratios and 95% confidence intervals for developing hypertension according to the maintenance of a low/high frequency of whole grain intake over the 3-year study period.
