# Supplementary material for: Prospective Association between Whole Grain Consumption and Hypertension: The Furukawa Nutrition and Health Study
Source: Nutrients. 2020 Mar 26;12(4):902. doi: 10.3390/nu12040902 (PMC7230178; doi:10.3390/nu12040902)
Supplement: Supplementary file 1 [file nutrients-12-00902-s001.pdf]

Table S1. Baseline characteristics of the participants who completed the follow-up survey versus those lost to follow-up.

	**Completed Follow-Up (*n* = 952)**	**Lost to Follow-up (*n* = 531)**	P_{trend} [1]
Whole grain consumption (sometimes or always), %	20.5	22.8	0.30
Age (mean ± SD), years	39.9 ± 8.6	40.9 ± 10.8	0.04
Females, %	11.7	11.7	0.99
Work site (April 2012 survey), %	53.4	54.4	0.69
Smoking status (current), %	30.5	27.5	0.23
Physical activity during work, housework, or commuting (≥ 20 MET-hours/day), %	23.1	19.2	0.08
Leisure-time physical activity (≥ 10 MET-hours/week), %	28.2	25.1	0.20
Body mass index (mean ± SD), kg/m^2	22.5 ± 2.9	22.7 ± 2.9	0.16
Night or rotating shift work (yes), %	20.1	16.0	0.05
Overtime work (≥ 30 hours/month) %	25.3	36.2	<0.001
Alcohol consumption (current drinker[2]), %	51.5	51.4	0.98
Dietary nutrient and food intake (mean ± SD)			
Total energy, kcal/day	1785 ± 478	1827 ± 510	0.12
Carbohydrates, % energy	55.0 ± 8.0	54.9 ± 7.7	0.81
Fat, % energy,	24.1 ± 5.5	24.4 ± 5.4	0.29
Protein, % energy	13.7 ± 2.4	13.9 ± 2.5	0.12
Sodium, mg/1000 kcal	2299 ± 450	2310 ± 494	0.66
Vegetables, g/1000 kcal	119.5 ± 65.7	118.8 ± 62.2	0.84
Fruits, g/1000 kcal	42.3 ± 47.3	46.6 ± 46.4	0.09
Pulses, g/1000 kcal	25.7 ± 19.6	23.3 ± 18.1	0.02
Meat, g/1000 kcal	39.6 ± 17.9	40.2 ± 17.9	0.52

Dairy, g/1000 kcal	51.1 ± 50.6	55.6 ± 48.7	0.10
Soft drinks, g/1000 kcal	45.6 ± 69.3	42.2 ±63.1	0.35
Rice, g/1000 kcal	184.1 ± 65.8	184.3 ± 71.0	0.95
Bread, g/1000 kcal	21.8 ± 17.9	21.3 ±16.9	0.56
Noodles, g/1000 kcal	43.4 ± 29.6	43.8 ± 29.2	0.83

Abbreviations: MET, metabolic equivalents. [1]The independent t test was used to compare continuous variables and the chi-square test to compare categorical variables. [2]Alcohol consumption at least one day per week.

Table S2. Odds ratios and 95% confidence intervals for developing hypertension according to the maintenance of a low/high frequency of whole grain intake over the 3-year study period.

	**Maintenance of Low Intake Frequency**		**Maintenance of High Intake Frequency**	
Participants (*n*)	401		112	
Cases (*n*)	42		4	
OR (95% CI), Model 1[1]	1.00	(Reference)	0.32	(0.11–0.92)
OR (95% CI), Model 2[2]	1.00	(Reference)	0.20	(0.05–0.73)

Abbreviations: CI, confidence interval; OR, odds ratio. [1] Model 1 adjusted for age (continuous), sex, and work site (survey conducted in April 2012 vs. May 2013). [2] Model 2 additionally adjusted for smoking (never-smoker, former smoker, current smoker of <20 cigarettes/day, or current smoker of ≥20 cigarettes/day), alcohol consumption (non-drinker, current drinker of 1–3 days/month, current drinker of <1, 1 to <2, or ≥2 servings (23 g ethanol)/day), physical activity during work, housework, or commuting (<3, 3 to <7, 7 to <20, or ≥20 metabolic equivalents (MET)-hours/day), physical activity during leisure time (0, >0 to <3, 3 to <10, or ≥10 MET-hours/week), body mass index (kg/m^2), night or rotating shift work (yes/no), overtime work (<10, 10 to <30, or ≥30 hours/month), total energy intake (kcal/day), and nutrient/food intakes (sodium, vegetables, fruits, pulses, meats, dairy, soft drinks, rice, bread, and noodles) expressed as energy densities.
